# Clinicopathological and Perioperative Outcome of Appendiceal Tumors: Case Review of 31 Patients

**DOI:** 10.51894/001c.13487

**Published:** 2020-10-30

**Authors:** Haroutioun Boyajian, Vanessa Majeski, Alicia Flores, David Sturtz, Fadi Baidoun, Mohammed Dughayli

**Affiliations:** 1 Surgery Henry Ford Wyandotte Hospital; 2 Pathology Henry Ford Wyandotte Hospital

**Keywords:** goblet cell, mucinous adenocarcinoma, mucinous cystadenoma, appendiceal tumors; carcinoid non carcinoid

## Abstract

**INTRODUCTION:**

Neoplasms of the appendix are quite rare and found in approximately 1% of appendectomy specimens. These neoplasms have been pathologically categorized into various subgroups depending on cell of origin, and surgical treatment varies according to histological subtype and disease stage.

**PURPOSE:**

The purpose of this case series review was to evaluate the clinicopathological presentation and survival outcome of a sample of patients with appendiceal tumors.

**METHODS:**

Before data collection, this project design was approved by the authors’ institutional review board. Pathology records at our institution were reviewed for cases of appendiceal tumors from January 2007 to December 2016. A total of 31 patients were identified over this 10-year period. Retrospective data collection included patient demographics, presenting symptoms, tumor size, histologic diagnosis, initial and secondary management, perioperative and postoperative outcome, and survival benefits.

**RESULTS:**

Thirty one patients with four different appendiceal tumor subtypes were included in the study: Mucinous Cystadenoma, Mucinous Adenocarcinoma, Goblet Cell, and Carcinoid. The sample was comprised of 17 women (54.8%) and 14 men (45.2%) with an overall mean age of 50.1 (SD = 22.3). Subgroups of 13 (42%) patients had Carcinoid tumor, 12 (39%) had Mucinous Cystadenoma, four (13%) had Goblet cell tumor, and two (6%) had Mucinous Adenocarcinoma. The stage at presentation and tumor size also varied by histologic subtype. The most common presenting symptom was abdominal pain (64.5%), followed by a radiological identified mass (12.9%). Overall, 27 (87.1%) patients survived, and four (12.9%) were deceased.

**CONCLUSIONS:**

The findings from this case series review provides a retrospective analysis of appendiceal tumor characteristics, follow up, and survival. Based on these results, the prognosis and management of patients with these tumors should be based on the histologic subtype and the extent of their disease.

## INTRODUCTION

Neoplasms of the appendix are rare and typically found incidentally in approximately 1% of appendectomies and are rarely suspected preoperatively.^1^ They account for 0.4% to 1% of all gastrointestinal malignant neoplasms.^2^ A 15-year review from the National Cancer Institute Surveillance Epidemiology and End Results (SEER) program found the age- adjusted incidence of appendiceal malignancies to be 0.12 cases per 1,000,000 people per year.^1^

The most common appendiceal tumors are epithelial neoplasms and neuroendocrine tumors (NET). Other tumors include lymphoma, metastasis, neuroectodermal, nerve sheath tumors, and mesenchymal tumors. The appendiceal epithelium contains enterocytes, goblet cells, and enterochromaffin cells, and lymphoid tissue which can give rise to epithelial tumors, Goblet Cell Tumor (GCTs) and NET, respectively.^3^ The appendiceal mucosa and submucosal layers have lymphoid tissue which can give rise to lymphomas.^3^

The histologic type of tumors predicts the biologic behavior and patterns of disease spread, is prognostic and impacts treatment.^4^ Patients with well differentiated appendiceal tumors with cells resembling normal cells, tend to grow and spread more slowly than poorly differentiated tumor cells. Patients with well differentiated tumors have been shown to have significantly improved survival.^5,6^

Carcinoid tumors are by far the most common, accounting for approximately 66%, with Cystadenocarcinomas accounting for 20% and Adenocarcinomas accounting for 10%.^1^ Rare forms of cancers include Adenocarcinoid (Goblet), Signet-Ring, Non-Hodgkins lymphoma, and Ganglioneuroma. Benign tumors include Adenomas, Cystadenomas, and benign Mucoceles. Appendiceal Adenocarcinoma occurs most frequently in middle aged or older adults, mostly in the fourth and fifth decades, although malignant NET are seen in younger patients in the second decade.^7^

Epithelial neoplasms of the appendix occur most commonly in the fifth through seventh decades of life.^7^ They are classified as mucinous and non-mucinous (colonic type).^8^ Mucinous neoplasms comprise 70% of the epithelial tumors.^9^ They are distinguished histologically into four types based on the depth of invasion of the wall, degree of atypia, and presence of mucin outside the lumen.^10^ These four types are named: adenomas, Low-Grade Appendiceal Mucinous Neoplasm (LAMN), High-Grade Appendiceal Mucinous Neoplasm (HAMN), and mucinous adenocarcinoma.

Appendiceal adenomas are confined to the mucosa and may have low-or high-grade atypia. LAMNs generally have low-grade atypia and one of the following features: loss of muscularis, fibrosis of submucosa, rupture of the appendix, and mucin and/or cells outside the appendix. HAMN have all the features of LAMN, but with high-grade atypia.^10^

Mucinous adenocarcinoma show infiltrative invasion (i.e., destructive pattern of invasion) and may be well, moderately, or poorly differentiated.^10^ These tumors can lead to Pseudomyxoma Peritonei (PMP), which is characterized by the presence of mucin and/or epithelial cells within the peritoneum and on the serosa of the abdominal and pelvic organs.^10,11^ The presence of PMP beyond the periappendiceal region indicates metastatic disease (M1a disease).^12^ Nonmucinous epithelial neoplasms comprise 30% of epithelial tumors.^9^ They include polyps, adenoma, and adenocarcinoma.^11^

Tumor staging is based on the National Comprehensive Cancer Network (NCCN) guidelines for colon adenocarcinoma.^13^ GCTs are infiltrative neoplasms, contain components of both adenocarcinoma and carcinoid, and are classified as mucinous or non-mucinous. NET tumors may also manifest with regional nodal involvement, or metastatic disease. The presence of signet-ring cells generally indicate a poor prognosis.^7^

During this case series report, the authors will present 31 patients with appendiceal tumors at their institution over a 10-year period, highlighting their clinicopathologic presentations, preoperative and postoperative outcomes, and subsequent survival outcomes based on different histologic types.

## METHODS

Before data collection, this study was approved by the Henry Ford Health System institutional review board. Using the hospital’s electronic health records (EHR), pathology reports were searched for appendiceal tumors from January 2007 to December 2016 as data prior to this time period was not available through the EHR database. A total of 31 eligible patients were identified. Data included demographics, presenting symptoms, initial management, histologic diagnosis, tumor size <=1cm, >1 to <=2cm and >2cm, and survival outcomes. No patients were excluded and all 31 patients diagnosed with appendiceal tumor were included in the case series review.

Demographic data and tumor characteristics were compared for each of the different histologies. A thorough and comprehensive chart review was performed by the authors, all patients’ pathology reports and their notes reviewed for additional surgical interventions and postoperative survival outcomes. Survival data were obtained from the patients’ electronic health records. All surviving sample patients were further contacted for additional information regarding any undocumented complications, (e.g., infection, surgical site hernia, and recurrence of cancer).

## RESULTS

A total of 31 patients with four different appendiceal tumor subtypes were identified to be included in the study- Mucinous Cystadenoma, Mucinous Adenocarcinoma, GCT, and Carcinoid. The sample comprised of 17 women (54.8%) and 14 men (45.2%). Overall mean age of the sample was 50.1 (SD = 22.3); the mean ages for patients with Mucinous Cystadenoma was 59.8 (SD = 21.8), 70 (SD = 7.1) years for patients with Mucinous Cystadenocarcinoma, 56.8 (SD = 21.9) years for patients with GCTs, and 36.1 (SD = 17.2) years for patients with Carcinoid. A total of 13 (42%) sample patients had Carcinoid tumor, 12 (39%) had Mucinous Cystadenoma, four (13%) had GCT, and two (6%) had Mucinous Adenocarcinoma. (Table 1)

Sample patients’ stage at presentation also varied by histologic subtype. Mucinous Cystadenoma and Carcinoid tumors had the highest proportions of localized disease at presentation, while Mucinous Adenocarcinoma and GCTs had the lowest. 10 (83.3%) patients with Mucinous Cystadenoma, 10 (76.9%) patients with Carcinoid, one (25%) patient with GCT, and none of the Mucinous Adenocarcinoma patients presented with localized tumor at time of diagnosis. Two (16.7%) patients with Mucinous Cystadenoma, three (23.1%) patients with Carcinoid, three (75%) patients with GCT, and one (50%) patient with Mucinous Adenocarcinoma presented with regional tumor at time of diagnosis. Only one (50%) patient with Mucinous Adenocarcinoma had a distant metastasis at the time of diagnosis. (Table 1)

Tumor sizes varied according to the type of appendiceal tumor. The majority of appendiceal tumors were small (<2 cm), contributing to the majority being detected incidentally after surgery. Nine (69.2%) patients with Carcinoid tumor had a tumor size of ≤ 1 cm while the remaining had a tumor size >1 cm. Six (50%) patients with Mucinous Cystadenoma had a tumor size of >2.1 cm, while three (25%) patients had tumor size ≤ 1 cm and the remaining three (25%) patients had tumor size 1.1-2cm. Both patients with Mucinous Adenocarcinoma had tumor size of >2.1 cm. Two (50%) patients with GCT had a size of ≤ 1 cm, one (25%) patient had a size 1.1-2cm and the remaining one (25%) patient had a size of >2.1cm. (Table 1)

**Table 1:** Demographic and Clinical Data for Appendiceal Histology

	Mucinous Cystadenoma	Mucinous Adenocarcinoma	Goblet Cell	Carcinoid
No. of cases	12 (39%)	2 (6%)	4 (13%)	13(42%)
Age	68	70	63	32
Male	2	1	3	2
Female	10	1	1	11
Tumor size average (cm)	1.6	3.2	3.8	1.2
≤ 1.0	3	0	2	9
1.1-2.0	3	0	1	3
>2.1	6	2	1	1
				
Overall Stage				
Localized	10 (83.3%)	0	1(25%)	10(76.9%)
Regional	2 (16.7%)	1 (50%)	3(75%)	3(23.1%)
Distant	0	1 (50%)	0	0

A total of 20 (64.5%) sample patients had presented to the emergency department with abdominal pain as their chief complaint. Four (12.9%) patients had a radiologically identified mass, three (9.7%) patients complained of nausea and vomiting, three (9.7%) patients had abdominal distention, and one (3.2%) patient had hematochezia (i.e., passage of fresh blood through the rectum and anus). As evident, the most common presenting symptom was abdominal pain, followed by a radiological identified mass. (Table 2)

**Table 2:** Frequency of Signs and Symptoms in Patients at Presentation:

Chief complaint	Frequency
Nausea and vomiting	3 (10%)
Abdominal pain	20 (64%)
Abdominal distension	3 (9%)
Hematochezia	1 (3%)
Radiologic finding of a mass	4 (13%)

### Survival outcomes

Each of the 31 sample patients were followed up from the time of surgery to April 2018. Twenty-three patients were followed up for more than five years, and eight patients were followed for less than five years. Of those eight patients with shorter follow-up periods, four were deceased and the remaining four were still alive. Overall, 27 (87.1%) patient survived, and four (12.9%) were deceased.

Of those 12 patients with Mucinous Cystadenoma, 10 (83.3%) were still alive after five years. (Tables 3 & 4). Seven were followed for greater than five years, and three patients were followed for 19, 28, and 48 months, respectively. Two patients were deceased; one was diagnosed with metastatic Mucinous Carcinoma of descending colon and was deceased at 42 months, and the other patient was deceased at 56 months. (Table 3) Of the 10 surviving patients, nine had localized tumor and one had regional metastasis. (Table 4)

**Table 3:** Clinical and Pathological Characteristics, Perioperative and Follow Up Data of Investigated Patients

	Age Range	Sex	Presentation	Surgical procedure	Morbidity	Resection margin	Tumor size	Pathology	Stage	Follow up duration (MO)	Recurrence
1	30s	F	Abdominal pain	Appendectomy	None	CRM	1.3	Carcinoid	l	>60	None
2	30s	F	Abdominal pain	Appendectomy R.C	None	CRM	3	Carcinoid	l	>60	None
3	40s	F	Abdominal pain	Appendectomy	None	CRM	5.5	Mucinous cyst adenoma	L	>60	None
4	70s	F	Adnexial mass	Appendectomy	Wound infection	CRM	4.5	Mucinous cystadenoma	L	>60	None
5	30s	M	Abdominal pain	Appendectomy	None	PRM	0.8	GCT	l	>60	NA
6	60s	M	Nausea and vomiting	Appendectomy	none	CRM	0.4	Carcinoid	l	>60	None
7	40s	M	Abdominal pain	Appendectomy R.C	None	CRM	3	GCT	R	>60	None
8	30s	F	Hematochezia	Appendectomy R.C	None	CRM	3.5	Carcinoid	R	>60	None
9	Teens	F	Nausea and vomiting	Appendectomy R.C	None	PRM	0.5	Carcinoid	L	>60	
10	30s	M	Abdominal distension	Appendectomy R.C	None	CRM	1.2	Carcinoid	l	>60	None
11	20s	F	Abdominal pain	Appendectomy	None	CRM	1	Carcinoid	l	>60	
12	70s	F	Abdominal pain	Appendectomy R.C	Cardiac Arrest	PRM Metastatic Carcinamatosis		Mucinous adenocarcinoma metastatic	NA	Deceased Post-op Day 2 due to cardiac arrest	NA
13	60s	F	Abdominal pain	Appendectomy R.C	SBO	CRM	1.5	GCT	R	>60	None
14	20s	F	Abdominal pain	Appendectomy	None	CRM	0.8	carcinoid	l	>60	
15	80s	M	Pelvic mass	Appendectomy	None	CRM	1	GCT	R	14^*^	
16	6s	M	Abdominal pain	Appendectomy R.C		PRM	NS	Mucinous cystadenocarcinoma	R	40- Deceased	Adenocarcinoma of duodenum
17	20s	M	Abdominal pain	Appendectomy	None	CRM	1	Mucinous cystadenoma	l	>60	None
18	80s	F	Adnexial mass	Appendectomy	None	CRM	1	Mucinous cystadenoma	l	>60	None
19	70s	F	Pelvic mass	Appendectomy TAH,BSO	None	PRM	1.2	Mucinous cystadenoma PMP	R	56- Deceased	None
20	30s	F	Abdominal pain	Appendectomy	None	CRM	1.2	Mucinous cystadenoma	L	>60	DPAM 4 years later
21	50s	F	Nausea and vomiting	Appendectomy RC	None	CRM	1.7	Carcinoid	R	>60	
22	60s	F	Abdominal pain	Appendectomy	None	CRM	0.4	Carcinoid	l	>60	
23	30s	F	Abdominal pain	Appendectomy	none	CRM	0.9	Mucinous cystadenoma	l	>60	
24	60s	F	Abdominal distension	Appendectomy	None	CRM	).8	Mucinous cystadenoma	l	>60	
25	70s	F	Abdominal pain	appendectomy	none	PRM	NS	Mucinous cystadenoma(ruptured)*	R	19^*^	NA
26	20s	F	Abdominal pain	Appendectomy	None	CRM	0.8	Carcinoid	R	>60	
27	70s	M	Abdominal distension	Appendectomy	None	CRM	1	Mucinous cystadenoma	l	42- Deceased	Metastatic mucinous carcinoma descending colon
28	20s	F	Abdominal pain	Appendectomy	None	CRM	0.15	Carcinoid	ll	>60	None
29	30s	F	Abdominal pain	Appendectomy	None	CRM	0.7	Carcinoid	l	>60	None
30	80s	F	Abdominal pain	Appendectomy	None	CRM	5.4	Mucinous cystadenoma	l	48^*^	
31	50s	F	Abdominal pain	Appendectomy	None	CRM	3.5	Mucinous cystadenoma	l	28^*^	

*Patients not achieved 60 months of follow up

**Table 4:** Overall Survival Outcomes for Appendiceal Histologies

	Mucinous Cystadenoma*	Mucinous Cystadenocarcinoma	Goblet Cell*	Carcinoid
All Stages	10 (83.3%)	0 (0%)	4 (100%)	13 (100%)
Localized	9	0	1	10
Regional	1	0	3	3
Distant	0	0	0	0
Total	12	2	4	13

* Of the 12 patients with Mucinous Cystadenoma, seven were followed for >5 years, three were followed for 19, 28, and 48 months, and two were deceased at 42 and 56 months. Of the four patients with Goblet Cell Tumor, three were followed for >5 years and one followed for 14 months.

Both patients with Mucinous Cystadenocarcinoma were deceased during the follow-up; one deceased on postoperative Day 2 due to cardiac arrest, and the second developed adenocarcinoma of the duodenum at 40 months and was deceased within two weeks of diagnosis. (Tables 3 & 4).

Four patients were identified with GCT. Three were followed for >5 years and one followed for 14 months. All four GCT (100%) patients were alive. (Tables 3 & 4).

A total of 13 patients were identified with Carcinoid tumor and all of them were followed for >5 years. Each of these 13 (100%) patients were alive at five-year follow-up. (Tables 3 & 4).

## DISCUSSION

During this case series, the authors evaluated the clinicopathological presentations and survival outcome of a sample of patients with appendiceal tumors who had been treated at our institution over a 10-year period. Appropriate treatment decisions require recognition of the different histopathological disease groups.^1^

These study results indicate from examination of the demographic and clinical data that carcinoid patients have significantly better survival than non-carcinoid patients. This outcome was expected since carcinoid tumors tend to be smaller (< 2 cm) with a lower risk of metastatic spread contributing to better survival rates compared to patients with non-carcinoid tumors.

Similar to other reports in the literature, a preoperative diagnosis of appendiceal neoplasms was rarely made for these case series patients.^14,15^ Most earlier reports indicate that most appendiceal neoplasms had been found incidentally after appendectomy and pathological examinations of the appendiceal tissue.^14,15^ Appendiceal neoplasms can present with variable presentations, but around 50% present as appendicitis and 0.7% to 1.7% are diagnosed on pathologic examination of the surgical specimen.^1^

These types of patients usually present to the emergency room, and initial evaluation is performed by emergency room staff, and afterwards, patients are evaluated by surgical teams. Appendiceal masses are sometimes also noted incidentally on abdominal CT scans. Benign tumors may be asymptomatic and discovered incidentally at pathologic evaluation after appendectomy, although malignant neoplasms may cause symptoms related to regional involvement, peritoneal spread, or metastatic disease.^8^

The most frequent initial manifestation of appendiceal tumors is acute appendicitis seen in 30%-to-50% of patients and more commonly in NET than in appendiceal neoplasms.^3,8,9^ Less frequently, symptoms include abdominal pain, a palpable mass, gastrointestinal bleeding, and abdominal distension, gastrointestinal obstruction. Frequency of symptoms in this case series is presented in Table 2. The most common presenting symptoms were nonspecific abdominal pain and abdominal distension. Often these patients are diagnosed with appendicitis before a neoplasm is found on their final pathology report, which resembles other reports in the literature.^14,15^

Earlier studies have shown that five-year survival for appendiceal tumors varies by tumor type. Patients with carcinoid tumors generally have the best five-year survival, whereas those with signet-ring cancers have the lowest.^16,17^ Mucinous histology has better survival than colonic histology for appendiceal adenocarcinoma,^18,19^ whereas Goblet histology demonstrates intermediate survival between carcinoid and adenocarcinoma.^20,21^

These case series results indicate that most tumors of the appendix will be non-carcinoids. There is a lot of variability in the recommendations for optimal treatment of non-carcinoid appendiceal tumors in the literature. For example, Lo and Sar^22^ demonstrated in 2003 a survival advantage for patients with mucinous adenocarcinoma treated with right hemicolectomy vs appendectomy. In 1995, Cortina et al^18^ reviewed 13 patients with adenocarcinoma of the appendix and found increased survival for patients treated with right hemicolectomy vs appendectomy, although the difference was not statistically significant.

Optimal treatment for epithelial mucinous neoplasms depends on the histological characteristics of the primary tumor and peritoneal disease, as well as the TNM stage.^13^ The treatment for adenoma and LAMN confined to the appendix is appendectomy. Cases of LAMN, with nodal involvement, HAMN, and adenocarcinoma without peritoneal extension are treated with appendectomy, right hemicolectomy and lymph node dissection.

Patients with peritoneal spread undergo laparoscopy.^4^ Based on the extent and histologic grade of their peritoneal tumors, cytoreductive surgery (CRS) and hyperthermic intraperitoneal chemotherapy (HIPEC) with the goal of complete resection of all visible tumor deposits greater than 2.5 mm^4,13,23^ Those patients who are not eligible for CRS should undergo systemic chemotherapy.^12^ The completeness of cytoreduction has been shown to correlate with survival.^19^

Patients with peritoneal disease should be followed up with CT imaging after CRS and HIPEC to detect PMP recurrence.^11^ Serum tumor markers carcinoembryonic antigen (CEA), CA19-9, and CA-125 are elevated in most patients with mucinous and non-mucinous epithelial tumors and advanced disease and correlates with treatment outcome.^4,16^

Non-mucinous appendiceal neoplasms include polyps, adenoma, and adenocarcinoma.^11^ Patients with tumors larger than 2 cm, nodal disease, or metastatic disease should undergo complete staging with CT of the chest, abdomen and pelvis.^12^ For tumors smaller than 2 cm without involvement of the appendiceal base and mesoappendix, some surgeons advocate appendectomy while others recommend right hemicolectomy.^12,17^

Right hemicolectomy (i.e., removal of the right side of the colon) is considered the standard of care for adenocarcinoma larger than 2 cm or involving the proximal appendix or in the presence of high-grade histologic features. Metastatic disease as well as node positive is an indication for systemic adjuvant chemotherapy.^12,13^ Patients first should undergo colonoscopy to evaluate for synchronous colorectal tumors.^13^

SEER data has showed the five-year survival rates for localized adenocarcinoma to be 95%, compared with a five-year survival of 80% for mucinous or cystadenocarcinoma. When distant metastasis were present, the five-year survival rates were 0% and 51% respectively.^4^

Staging of GCTs should be performed with CT of the chest, abdomen and pelvis. The most common site of metastasis is the peritoneum.^14^ Simple appendectomy is recommended for GCT even though it is an aggressive tumor. However, right hemicolectomy and lymphadenectomy are performed in cases of cellular undifferentiation, increased mitotic activity, involvement of the base of the appendix tumor greater than 2 cm, and lymph node metastasis.^24^

Nodal involvement is an indication for adjuvant chemotherapy, peritoneal metastasis is treated with CRS and HIPEC. CEA, CA19-9, CA-125 are useful post-treatment follow up. GCT have the worst prognosis and are more aggressive than carcinoids, although they are not as malignant as adenocarcinomas. The five-year survival rate is 81%,^7^ and long-term surveillance for disease recurrence is recommended.

Appendiceal NET may manifest with regional nodal involvement or metastatic disease. Carcinoid syndrome is seen in less than 5% of NET and most commonly related to liver metastasis.^25,26^ Tumor size at diagnosis correlate with nodal or distant metastatic involvement. 95% of patients with tumors less than 2cm rarely present with metastasis.^24^

When NET is discovered incidentally at appendectomy, CT imaging of chest and pelvis is recommended postoperatively if the T is larger than 2 cm, has positive margins at resection, or associated with metastatic disease.^4^ TNM staging of appendiceal NET is different from that of gastrointestinal NET. The extent of surgery is based upon the size of the tumor. Since most carcinoid tumors are found incidentally on simple appendectomies, a second surgery is sometimes needed. Tumors smaller than 1 cm are treated with simple appendectomy, while tumors larger than 2 cm require right hemicolectomy and regional lymphadenectomy.^4^

The appropriate resection for masses between 1 and 2 cm is controversial.^13^ Guidelines from North America Neuroendocrine Tumors Society (NANETS) recommend right hemicolectomy for tumors between 1 and 2 cm in the presence of deep mesoappendiceal invasion, positive or unclear margins, higher proliferative rate, angioinvasion and mixed histologic features.^13^ Before the procedure, colonoscopy should be performed to rule out synchronous colon cancer. In the setting of locoregional spread or distant metastasis, treatment depends on whether the primary tumor/ or metastasis can be resected.^13^

The follow up of patients with NETs depends on the size of the tumor and is based on the National Comprehensive Cancer Network (NCCN) guidelines.^13^ For tumors less than 2 cm treated with appendectomy, no routine surveillance is indicated. For tumors greater than 2 cm, those with nodal involvement, or those treated with right hemicolectomy, a history, physical examination and measurement of serum cytogranin A (CGA) level are recommended three to 12 months after resection with consideration of CT or MRI.^13^

Starting the first year after surgery, a history and physical examination every six to twelve months, along with serum CGA testing, and CT or MRI is generally recommended as standard of care.^27^ This surveillance should continue up to 10 years. Overall five-year disease- specific survival rates for appendiceal NETs approach 85-100% when the tumor is confined to the appendix, and 34% in patients with distant metastasis.^14^

In our case series, the surgical procedures performed by tumor type, tumor size, and extent of the disease generally matched the current recommendations in the literature. Noncarcinoid appendiceal tumors and carcinoid tumors greater than 2 cm required more aggressive treatment. In our series, both patients with carcinoid tumors >2 cm received right hemicolectomy and lymphadenectomy and were alive at five years. Four patients with mucinous cystadenoma >2 cm (localized to the mucosa) were treated with appendectomy and were alive at five-year follow up. One patient had GCT treated with appendectomy and colectomy and was alive at five years. There were four non-carcinoid patients treated with appendectomies only because the tumor was localized to the mucosa.

Although hemicolectomy is advised for GCTs, some authors have reported that low-risk tumors <1 cm, localized tumors without serosal, mesoappendiceal, or cecal invasion, with low proliferative index can be better served with appendectomy alone.^28,29^ Following these criteria, only one of our patients with GCTs did not follow these recommendations although they were found alive at five year follow up.

There were several limitations to this case series. The data were derived from a smaller community-based convenience patient sample. Patients’ comorbidities were not analyzed, nor were initial indications for surgery always clearly reported (e.g., appendicitis versus an incidental finding on CT imaging). Available information regarding adjuvant chemotherapy or radiation therapy was also limited.

Finally, many of these patients did not complete periodic clinical follow-up throughout their five postoperative years, and some important information could have been better gathered from phone calls (i.e., based on patient recall).

## CONCLUSIONS

These study results indicate that epithelial neoplasms and NET comprise the majority of appendiceal tumors. It is evident from these findings that a substantial number of patients in the two categories, carcinoid and noncarcinoid tumors of the appendix, and may not be receiving appropriate surgical resection.

Based on these findings, there remains a need for considered decision making and follow up by multidisciplinary teams of surgical personnel and primary care physicians to ultimately improve survival of these patients.

The authors report no external funding source for this study.

The authors declare no conflict of interest.

Submitted for publication April 2020.

Accepted for publication June 2020.
